# A Case of Narcolepsy
1Read at a meeting of the Society, December 12th, 1906.


**Published:** 1907-06

**Authors:** Bertram M. H. Rogers

**Affiliations:** Physician to the Royal Hospital for Sick Children and Women, Bristol.


### A CASE OF NARCOLEPSY.[1]

BY

Bertram M. H. Rogers, M.D. Oxon.,

*Physician to the Royal Hospital for Sick Children and Women, Bristol.*

---

By narcolepsy I wish you to understand a condition in which a patient, with almost lightning-like rapidity, falls into a sleep of short duration, the condition not being one of epilepsy.

The patient in whom I observed this condition was a young lady, unmarried, of about 30 years of age. She comes of a highly neurotic stock, chiefly, however, on the mother's side. In the maternal family there are two brothers, one of whom took his own life, while the other is a confirmed dipsomaniac. In a married sister's family there is a daughter in an asylum, a son with disseminated sclerosis, another son who wanders about the world unable to settle to any definite work, but always expecting to make a fortune by some hare-brained scheme, such as a mule service between Suakim and Khartoum; another sister died of Addison's disease, and a son, after an unfortunate circumstance connected with the firing of big guns, became a hopeless nervous wreck and shot himself. In the patient's own family there are no such strong evidences of the neurotic taint, but her sisters are all of a mild neurotic type, requiring rest cures at various times for conditions produced by slight mental stress. The general health of all is good, though not robust, and their mental activity is good, all taking part in social and some in philanthropic work.

The subject of this paper is, perhaps, the most intelligent of the daughters. She has attended various courses of lectures on science or literature, takes in what she hears, and has composed short stories and plays of quite a fair standard of excellence. They are not brilliant literary efforts, but I wish to point out that her intellectual powers are not deficient; on the contrary, she is actively and constantly employed in some mental exercise,

[1] Read at a meeting of the Society, December 12th, 1906.

of a nature rather above the usual standard of young women who have not to earn their own living. Her general health is good, menstrual functions normal, and her visual defects have been corrected with glasses.

The first symptom noticed and complained of was occipital headache. She told me that it seemed as if someone was attempting to lift her up by her back hair. I may mention that she has a great profusion of hair: not only is it long, but extraordinarily thick; in fact, it must seem to some of us who have little of that ornament that the amount of hair many women carry must be of great weight, especially when the arrangement of it is assisted by puffs of various kinds. These headaches as a rule came on in the morning, soon after or at waking, and lasted till midday, when they disappeared. They were very intense while they lasted, necessitating a darkened room and absolute quiet in bed. There never was any vomiting, and the retinæ presented no changes. After the headache, the patient was quite well for the rest of the day; could go out for a walk, or bicycle a short distance, though a greater effort might bring on a return of the head trouble. After a week or two of almost daily headaches sudden short lapses into sleep began. What I observed was that on talking to her she suddenly stopped talking, her eyelids slowly closed after twitching slightly or screwing up of the lids, her hand went to the back of her head, the movement being as if she was in pain, and then with a slight turn of her head to the right she was fast asleep. After a few minutes, or even less at times, she opened her eyes, blinked a little, and went on with the conversation where she left off, seldom if ever losing the thread of her conversation. On waking she was sometimes rather excited in manner, but was evidently quite ignorant of her lapse into sleep. This sudden falling asleep might take place two or three times in the quarter of an hour or more that I saw her, sometimes in the middle of writing or drawing something, or even while eating. This, I was told, would continue all the morning, sometimes with headache, sometimes without, but nearly always she had a heavy sleep for an hour or two in the afternoon. By the evening she had nearly thrown off the condition, but might occasionally go off suddenly while at dinner. She slept well, almost too heavily, at night. After a few weeks of this condition she began to lose flesh, and her digestion got out of order. Not unnaturally, her parents got rather alarmed, not only about her present state, but for the future, and though I was able to assure them that there was no evidence of serious mischief at the time, I was rather anxious for the future. After a couple of months of varying degrees of somnolence she began to improve, the first symptom that began to give way being the headaches: these got less in intensity and in frequency. Her parents then took her to London to see a distinguished psychologist, but unfortunately she slept and could not be aroused the whole time he was there. A short time before going to London a new symptom began to show itself—pain in the left iliac region, with a sort of spasm when the somnolent state came on. A change of treatment was suggested by the London physician, but did not appear to make much difference, the trouble slowly but surely improving. When her condition allowed her to be about more, she went abroad with a sister and nurse for three months, returning quite well, having lost all pain, all tendency to sleep at wrong times, and having put on flesh to her normal condition.

The patient continued quite well for over two years, went about in society without anyone noticing that anything had been the matter, and resumed her ordinary occupations and studies, in which she showed considerable aptitude, following them with diligence and with considerable intelligence. In fact, apart from the inherent neurotic condition, she was quite well.

The liability to narcolepsy was not, however, entirely eradicated, and, as I shall show, was only dormant, for it was reawakened by a trivial circumstance on one occasion, and by a severe mental stress again some years after.

The first recurrence followed a slight operation which was necessary to relieve an inflamed gland in the neck. She took the anæsthetic well, and recovered from it without any trouble, but on the following day the nurse told me that she had on several occasions lapsed into sleep of short duration, a few minutes or so, to wake up quite unconscious of having lost herself. This attack was of exactly similar character to the former one, but unaccompanied by headache, and of a decidedly milder type. On more than one occasion she dropped off while the surgeon and I were in the room, waking up suddenly and continuing the conversation where she had ceased before her sleep. This attack lasted only a few days, but naturally caused both the family and myself considerable anxiety as to whether the condition would improve soon, or whether the patient was in for a long period of liability to the attacks as before. Before the wound healed—a week or so—she was quite well, and the symptoms of the condition passed off.

The second relapse followed, as I said, after a severe mental strain. The patient's mother, to whom she was exceedingly attached, died after a few days' illness away from home at a seaside resort. The narcoleptic condition only presented itself once or twice. I only saw her once pass into the semi-conscious state not amounting to actual sleep. She was sitting in a chair, when I observed her close her eyes and appear to lose her balance for a few moments, recovering with the blinking of the eyes that I noticed in the first attack. Had I not known and seen the first attack, I should hardly have recognised what the nature of it was; perhaps I might have thought it a passing fainting attack, but, knowing her previous history, I have no doubt that she had a slight recurrence.

Since this time she has been quite well, and uses her brain considerably for work of all kinds, and as much as she has ever done.

I have no intention of entering any further into a discussion, or giving you an essay on this condition. A very large number of cases have been reported in medical literature differing slightly in details, but in the main the same as the case I have just read. They are interesting cases. The conditions that manifest themselves are so peculiar and strange, and the causation and pathology so obscure, that we cannot hope to be able to explain the phenomena without further observation.

I wish only to point out that the symptoms are not in any way allied to epilepsy, a fact which writers on this condition lay special emphasis, nor is the relationship to normal sleep quite clear. Raymond, who has written on the subject, in alluding to the pathological varieties of sleep, "mentions a condition which would seem to represent the border-line between normal sleep and the unconsciousness often accompanying recognisable lesions of the cerebral structures," and perhaps this "border-line,"—a word I do not at all like—may be taken to be held by this condition known as narcolepsy. He recognises a mental hebetude and abnormal slumberous conditions associated with disordered mental activity.

Raymond's account might have been taken from my patient. He describes the condition as one in which the patient outside the usual hours of sleep passes into slumber. The sleep comes on suddenly in the midst of ordinary occupations, even, he says, as in my case, during meals. He states it is noticed in subjects liable to gout, rheumatism, or obesity, or suffering from auto-intoxications. Dr. Blodgett's patient had been subject to the attacks continuously for forty years, and often had several attacks in one day. She fell into a perfectly natural sleep, and could be easily roused by a call or an unusual noise, waking up unaware that she had been asleep, though at times the state was more profound, and she realised that she had passed an appreciable interval in unconsciousness. In spite of these numerous attacks lasting over such a long time, very little mental deterioration was noticed by her friends, and it must be observed that the patient was reaching an age when some failure might not unnaturally follow.

Other writers remark on the association of this condition with a toxic poisoning—toxic poisoning is almost as blessed a word as Mesopotamia—with obesity or diabetes, or deem the association of these diseases as accidental or following the same causative action; but I am inclined to accept Dr. Blodgett's remark, "No definite information upon this causation of symptoms in narcolepsy is yet available." This gentleman, in the account of his case, lays considerable stress on the neurotic history of his patient's family. In this his case agrees with mine, for in both there are the frequent manifestations of some neurosis taking one form or another.

